# ciRs-6 upregulates March1 to suppress bladder cancer growth by sponging miR-653

**DOI:** 10.18632/aging.102525

**Published:** 2019-12-10

**Authors:** Yinjie Su, Weilian Feng, Guanglei Zhong, Yiyao Ya, Zehu Du, Juanyi Shi, Luping Chen, Wen Dong, Tianxin Lin

**Affiliations:** 1 The Department of Urology, Sun Yat-Sen Memorial Hospital, Sun Yat-Sen University, Guangzhou, China; 2 The Department of Endocrinology, Sun Yat-Sen Memorial Hospital, Sun Yat-Sen University, Guangzhou, China; 3 The Department of Gynecological oncology, Sun Yat-Sen Memorial Hospital, Sun Yat-Sen University, Guangzhou, China; 4 The Department of Urology, Guangzhou First People's Hospital, School of Medicine, South China University of Technology, Guangzhou, China; 5 The Department of Thyroid Surgery, Sun Yat-Sen Memorial Hospital, Sun Yat-Sen University, Guangzhou, China; 6 The Department of Pediatric Surgery, Sun Yat-Sen Memorial Hospital, Sun Yat-Sen University, Guangzhou, China

**Keywords:** ciRs-6, March1, bladder cancer

## Abstract

Background: Circular RNAs have been widely explored as potential biomarkers and therapeutic targets in bladder cancer; however, few have been functionally characterized.

Results: ciRs-6 is expressed at low levels in cancer tissues and advanced tumor grades and stages, and its expression correlates with better outcomes for bladder cancer patients. In vitro and in vivo, ciRs-6 was shown to suppress bladder cancer growth by sponging miR-653 to elevate March1 levels. March1 is an E3 ubiquitin ligase that has been proven to suppress bladder cancer growth; knocking down March1 in ciRs-6 overexpressed bladder cancer cells reversed the tumor suppressive effect of ciRs-6.

Conclusions: Our study identifies an oncogenic role of ciRs-6 and suggests its usefulness as a novel biomarker for bladder cancer diagnosis and prognosis and as a therapeutic target for bladder cancer.

Methods: ciRs-6 was identified by RNA-seq and qPCR; CCK8 assays, clone forming assays and cell cycle analyses were performed to evaluate the in vitro effect of ciRs-6 in bladder cancer; further, a mouse subcutaneous tumor model was designed for in vivo analysis. RNA pulldown assays, miRNA capture experiments and dual luciferase assessments were applied for mechanistic studies.

## INTRODUCTION

Despite multiple therapeutic options for bladder cancer, including surgery, BCG (bacille calmette-guerin) perfusion or chemotherapy, rapid growth and frequent metastatic potential in highly advanced cancers greatly limit clinical treatments [1]. Cancer progression is the result of various behaviors, including proliferation and metastasis. Hampering each process enables limiting or reversing malignancy, which is the basic principle of current chemotherapy; however, the effects are not ideal [2]. Among various possible contributors to poor results is the fact that individual differences result in different sensitivity and effectiveness of conventional treatments. Hence, it is necessary to further explore oncogenic signaling, which will better guide clinics regarding making comprehensive and personalized decisions for bladder cancer patients.

Malignant signals, including genetic and epigenetic signals, depend on the expression and regulation of intracellular genes. Compared with genetic factors, epigenetic variation does not involve altering genomic sequences, but this variation easily causes individual differences. Noncoding RNAs constitutes a major part of epigenetic regulation, and circular RNAs have recently been further explored as a popular RNA that promotes cancer [3]. ciRS-7 negatively regulates bladder cancer proliferation by activating p21 [4]; hypoxia elevates circELP3 to promote bladder cancer progression and drug resistance [5]; the circular RNA CEP128 promotes bladder cancer cell propagation and migration by regulating MAPK signaling [5]; and circHIPK3 decreases lung metastasis through suppressing heparanase expression in bladder cancer [6]. Because of the advantages of a ring structure, circular RNAs are able to resist various kinds of exonuclease, making them more stable than any other noncoding RNAs [7]. This stability is especially reflected in its role as a biomarker for predicting pathological and prognostic features in cancer. For example, circPRMT5 is significantly upregulated in serum and urine exosomes in bladder cancer patients, and its levels statistically correlates with tumor metastasis [8]. circ-ITCH [9], circHIPK3 [6] and circMTO1 [10] were all found to be decreased in bladder cancer tissues, and their levels negatively correlate with grade, stage, invasion and lymph node metastasis in bladder cancer patients.

In this study, we reanalyzed a circular RNA that we previously identified by mRNA-sequence analysis [5]. Among the various downregulated circular RNAs in bladder cancer tissue, ciRs-6 is one of the top ten and is positively associated with better outcomes in bladder cancer patients. In a series of in vitro and in vivo experiments, it was found that ciRs-6 could suppress bladder cancer growth by sponging miR-653 to elevate the levels of March1, which is a tumor suppressor gene. Generally, our study sheds new light on the biology of bladder cancer and identifies a novel potential biomarker that could be used for the detection and prediction of bladder cancer.

## RESULTS

### ciRs-6 is significantly downregulated in bladder cancer, and its expression is correlated with prognosis

**Figure 1:**
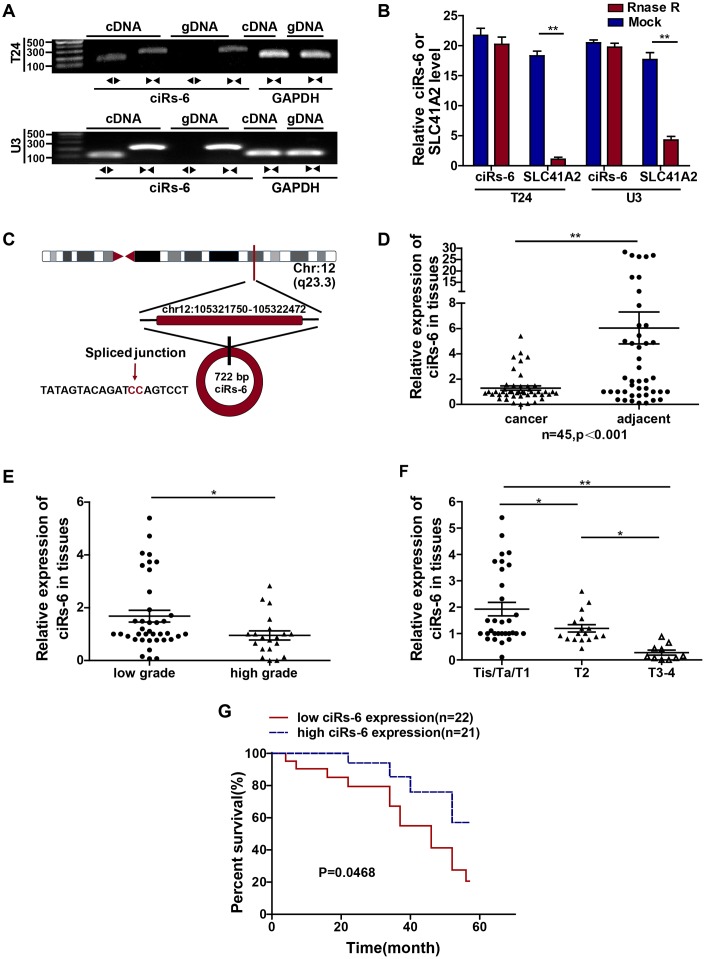
**ciRs-6 is downregulated in bladder cancer, and its downregulation is associated with poor prognosis**. (**A**) Divergent (◀▶) and convergent (▶◀) primers were designed for ciRs-6 (◀▶) and its linear form, SLC41A2 (▶◀), respectively. Agarose gel nucleic acid electrophoresis was used to detect both the cDNA and gDNA forms of ciRs-6 and SLC41A2. (**B**) RNase R treatment was used to evaluate the exonuclease resistance of ciRs-6. (**C**) The diagram schematically illustrates the circulation of ciRs-6. (**D**) qPCR was used to detect the expression of ciRs-6 in 45 paired bladder cancer tissues and adjacent normal tissues. (**E**) The expression of ciRs-6 in 58 bladder cancer tissues was evaluated by qPCR and analyzed for high/low grade and (**F**) T stages. (**G**) Relationship between overall survival and ciRs-6 level in bladder cancer patients was analyzed by Kaplan-Meier curves and log-rank tests. The median value of the ciRs-6 level was used as the cutoff. The results are displayed as the mean±SEM, *p<0.05, **p<0.01.

ciRs-6 was first identified according to the specific properties of circular RNAs: generation through back-splicing events and exonuclease resistance. By performing agarose gel electrophoresis, it was found that divergent primers can amplify ciRs-6 from cDNA but not genomic DNA (Figure 1A); in addition, after treating with RNase R, the linear form of ciRs-6, SLC41A2, was apparently digested, while the expression of ciRs-6 remained stable (Figure 1B). Furthermore, RNA Sanger sequence analysis was performed, and the sequences that were amplified by divergent primers were the same as the sequences of hsa_circ_0006260 in http://www.circbase.com (Figure 1C). These results suggest that this molecule is circular RNA. Later, we explored the clinical value of ciRs-6 in bladder cancer, and we observed a strong downregulation of ciRs-6 in bladder cancer tissues; ciRs-6 expression was 4.7-fold lower in 45 paired bladder cancer tissues than adjacent normal tissues (Figure 1D). Moreover, 58 bladder cancer patients were analyzed as well. Higher levels of ciRs-6 consistently correlated with lower tumor grade (Figure 1E), and the expression of ciRs-6 decreased from Tis/Ta/T1 to T2 and from T2 to T3-4 (Figure 1F). Moreover, in a cohort of 43 bladder cancer patients, a higher level of ciRs-6 in bladder cancer positively correlated with better overall survival, as shown with a Kaplan-Meier’s survival curve (Figure 1G). Taken together, these results suggest that downregulated ciRs-6 in bladder cancer is associated with better pathology and prognosis. The relationship between ciRs-6 level and clinical characteristics is shown in Table 1. The median was used to differentiate between high/low expression of ciRs-6.

**Table 1:** Relationship between ciRs-6 level and clinical characteristics in bladder cancer.

**Total**	**Patients**	**Expression of ciRs-6**		
		**High**	**Low**	**p**
**Age(mean)**	53.21	50.84	51.49	0.597
**Gender**				
Male	46	27	19	0.536
Female	12	7	6	
**Tumor stage**				
Tis/Ta/T1	22	17	5	<0.01
T2	17	11	6	
T3/T4	9	4	5	
**Grade**				
High	40	18	22	0.015
Low	18	3	15	
**Number of tumors**				
Solitary	41	22	19	0.674
Multiple	17	10	7	
**Lymph node metastasis**				
Negative	23	12	11	0.729
Positive	35	18	17	
**Follow-up (month, mean)**	33.23	33.45	34.62	0.046

### ciRs-6 suppresses the growth of bladder cancer cells

**Figure 2:**
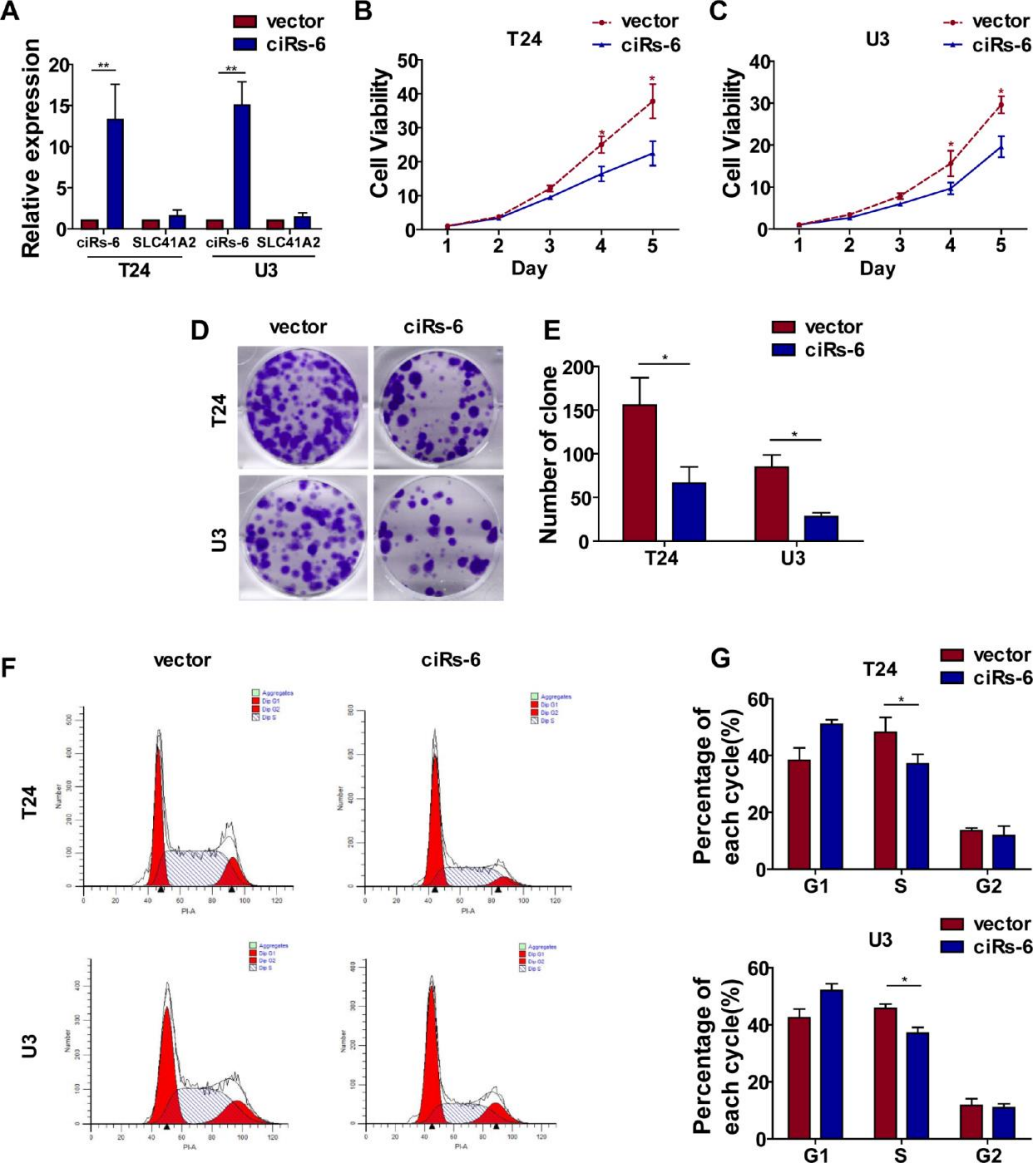
**Overexpression of ciRs-6 suppresses bladder cancer growth in vitro and in vivo**. (**A**) The levels of ciRs-6 and SLC41A2 in ciRs-6 overexpressed bladder cancer cells were determined by qPCR; (**B**, **C**) CCK8 assays were performed to evaluate cell viability; (**D**, **E**) clone formation assays were used to evaluate the ability to form clones; (**F**, **G**) cells in the S phase were assessed by cell cycle analysis; (**H**–**J**) mouse subcutaneous tumor model was used to detect the suppressive role of ciRs-6 in tumor growth in vivo. Each group contained 5 mice. The results are displayed as the mean±SEM, *p<0.05, **p<0.01.

Cell proliferation and metastasis are 2 main characteristics of cancer progression. To detect the role of ciRs-6 in bladder cancer, we first designed 3 siRNAs, and their efficacy in silencing ciRs-6 expression was determined by qPCR. It was found that all 3 siRNAs could silence ciRs-6 without effecting SLC41A2 (Supplementary Figure 1A). We analyzed proliferation as the first step. It was found that bladder cancer viability was significantly improved when cells were treated with siRNAs (Supplementary Figure 1B, 1C), and the clone forming assay showed increased clone formation as well (Supplementary Figure 1D, 1E). Moreover, cells were stained for cell cycle analysis, and there was an elevated percentage of cells in S phase following siRNA treatment (Supplementary Figure 1F–1H). These results suggested that silencing ciRs-6 promotes proliferation of bladder cancer cells. However, there was no obvious phenotype to show the role of ciRs-6 in cancer metastasis, as demonstrated by in vitro transwell and wound healing assays (Supplementary Figure 2A–2C). Moreover, stable bladder cancer cell lines overexpressing ciRs-6 were constructed, and the efficacy of overexpression was determined by qPCR (Figure 2A). Similar to the assays mentioned before, a lower cell viability (Figure 2B, 2C), clone formation ability (Figure 2D, 2E) and percentage of cells in S phase (Figure 2F, 2G) were observed when ciRs-6 was overexpressed in bladder cancer cells. Moreover, to prove that this reduced cell growth was due to changes in apoptosis, we performed an Annexin V/PI assay. The results showed that overexpression of ciRs-6 in bladder cancer cells led to less apoptosis (Supplementary Figure 5B, 5C). In a subcutaneous tumor xenograft model, there was a significant positive effect of tumor suppression when ciRs-6 was overexpressed (Figure 2H), as indicated by a decrease in tumor volume (Figure 2I) and weight (Figure 2J). In conclusion, the results above suggest that ciRs-6 suppresses bladder cancer growth.

### ciRs-6 suppresses bladder cancer growth by sponging miR-653

**Figure 3:**
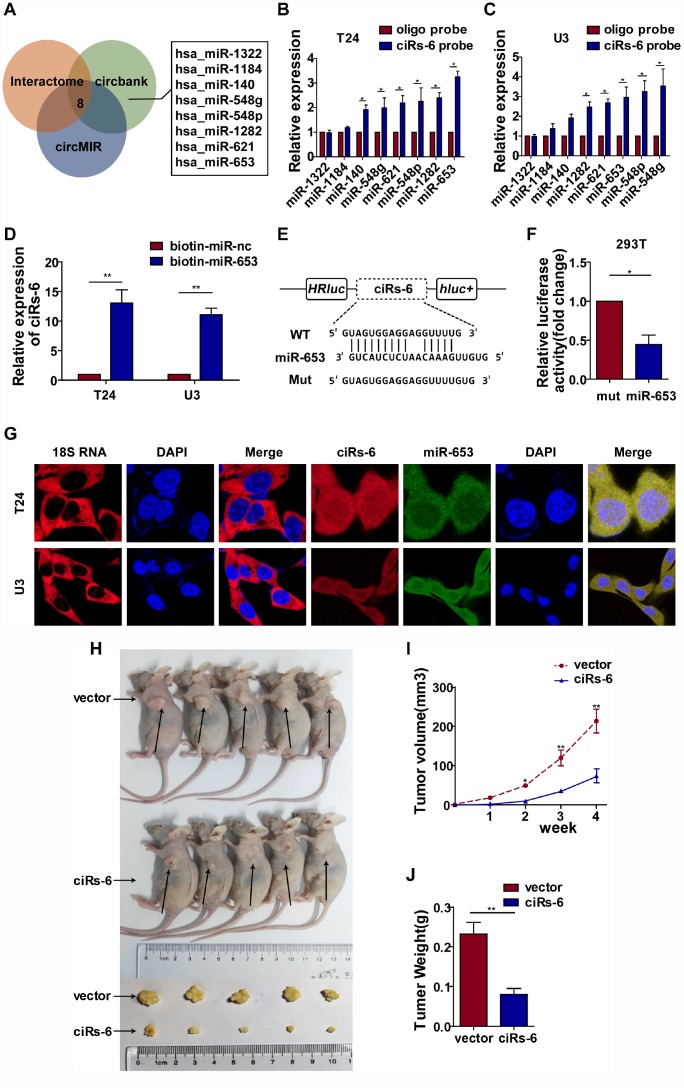
**ciRs-6 effectively sponges miR-653.** (**A**) miRNAs that could bind to ciRs-6 were predicted by Interactome, circBANK, and circMIR. Finally, 8 miRNAs were found commonly in each database; (**B**, **C**) RNA pulldown assays were performed to examine the affinity of ciRs-6 for the 8 above miRNAs in bladder cancer cells; (**D**) RNA pulldown assays were performed to evaluate the affinity of ciRs-6 for miR-653 in bladder cancer cells; (**E**, **F**) dual luciferase reporter assays showed binding properties of ciRs-6 and miR-653; (**G**) RNA FISH showed the cellular location of ciRs-6 and miR-653. Nuclei were stained with DAPI. Pictures were photographed at 400× under a light microscope. The results are displayed as the mean±SEM, *p<0.05, **p<0.01.

Subcellular localization plays a role in the function of molecules. Taking the nuclear membrane as a boundary, molecules inside the nucleus are mostly thought to function prior to gene transcription, while molecules outside are thought to work posttranscriptionally. Hence, we designed a probe that was specific for ciRs-6, and FISH assays showed that ciRs-6 was primarily present in the cytoplasm of bladder cancer cells (Figure 3G); this conclusion was supported by the expression of ciRs-6 being higher in the cytoplasm than the nucleus, as shown by nuclear and cytoplasmic extraction assays (Supplementary Figure 3D). These results suggest that ciRs-6 works posttranscriptionally. According to current published research, the main mechanistic functions of cytoplasmic circular RNAs are thought to be in acting as competing endogenous RNAs, especially sponging miRNAs. Considering all predicted miRNAs from Interactome, circBANK and circMIR, only 8 miRNAs overlapped and abled to bind ciRs-6 (Figure 3A). Next, an RNA pulldown assay showed that there were 5 miRNAs, miR-548g, miR-548p, miR-621, miR-1282 and miR-653, that were pulled down by a ciRs-6 probe in both bladder cancer cell lines (Figure 3B, 3C). To further narrow down the miRNAs targets of ciRs-6, we performed CCK8 and clone formation assays, and only miR-653 displayed a strong cancer promoting effect (Supplementary Figure 3A–3C). These results suggest that miR-653 might be the downstream binding factor of ciRs-6. Furthermore, we designed biotin-labeled miR-653, and ciRs-6 was found to associate with it more than with a biotin-labeled miR-nc (Figure 3D); moreover, a dual luciferase reporter assay showed an obvious reduction in luciferase activity when miR-653 and a reporter that contained a ciRs-6 binding site were cotransfected into cells (Figure 3E, 3F). Further, strong colocalization of ciRs-6 and miR-653 was observed in FISH assays (Figure 3G) and nuclear and cytoplasmic extraction assays (Supplementary Figure 3D) using bladder cancer cells; the expression of ciRs-6 and miR-653 did not change when both were overexpressed individually in bladder cancer cells (Supplementary Figure 3E, 3F). Hence, these results indicate that ciRs-6 sponges miR-653 in bladder cancer cells.

**Figure 4:**
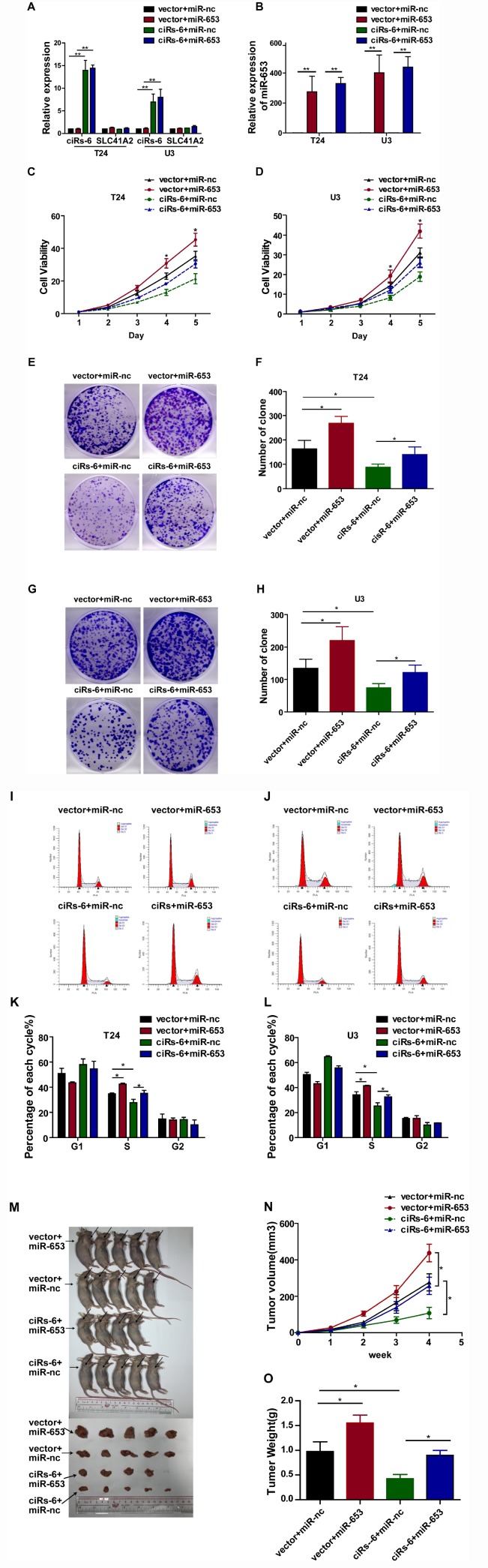
**miR-653 rescues the tumor suppressive effect of ciRs-6 in bladder cancer.** (**A**, **B**) qPCR was used to detect the levels of ciRs-6, SLC41A2 and miR-653 in each group; (**C**, **D**) CCK8 assays were performed to evaluate cell viability; (**E**–**H**) clone formation assays were used to evaluate clone forming ability; (**I**–**L**) cells in S phase were assessed by cell cycle analysis; (**M**–**O**) mouse subcutaneous tumor model experiments were used to detect in vivo tumorigenic effects. Each group contained 5 mice. The results are displayed as the mean±SEM, *p<0.05, **p<0.01.

To further evaluate how ciRs-6 sponging of miR-653 resulted in a tumor suppressive effect, a series of rescue assays were developed. Through CCK8 assays, clone forming assays, S phase analysis and in vivo assessment with a subcutaneous tumor model, miR-653 was found to significantly promote cell viability (Figure 4C, 4D), clone formation (Figure 4E–4H), the percentage of cells in S phase (Figure 4I–4L) and tumor formation in vivo (Figure 4M–4O). However, when miR-653 was co-overexpressed with ciRs-6 in bladder cancer cells, either the tumor suppressive role of ciRs-6 or the oncogenic role of miR-653 was reversed, making the results similar to those of wild type results in vitro and in vivo (here, as indicated by vector-treated cells) (Figure 4A–4O). Hence, these results suggest that ciRs-6 suppresses bladder cancer growth by sponging miR-653.

### ciRs-6 suppresses bladder cancer growth by elevating March1

**Figure 5:**
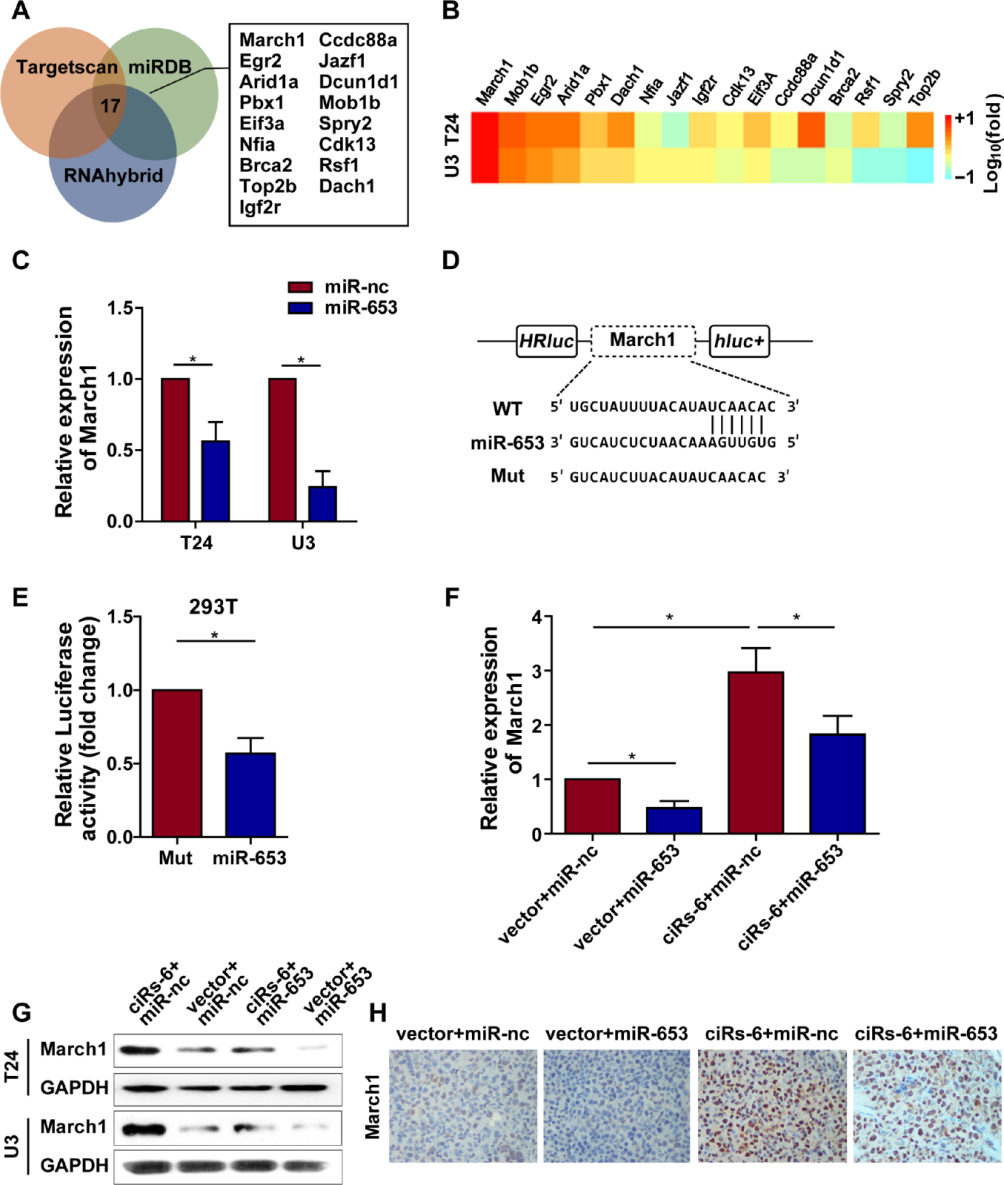
**ciRs-6 sponges miR-653 to elevate March1 levels**. (**A**) Target genes that binds to miR-653 were predicted by TargetScan, RNAhybrid, and miRDB; (**B**) qPCR was used to detect the expression of the 17 predicted targets following ciRs-6 overexpression; (**C**) miR-653 significantly reduced March1 expression in bladder cancer cells; (**D**, **E**) dual luciferase reporter assays showed binding between March1 and miR-653. (**F**, **G**) qPCR and Western blot experiments were performed to detect March1 levels among ciRs-6+miR-nc, vector+miR-nc, ciRs-6+miR-653, and vector+miR-653 cells. (**H**) IHC was performed to detect March1 levels in tissues that were previously collected from a subcutaneous tumor model. Pictures were photographed at 200× under a light microscope. The results are displayed as the mean±SEM, *p<0.05, **p<0.01.

**Figure 6:**
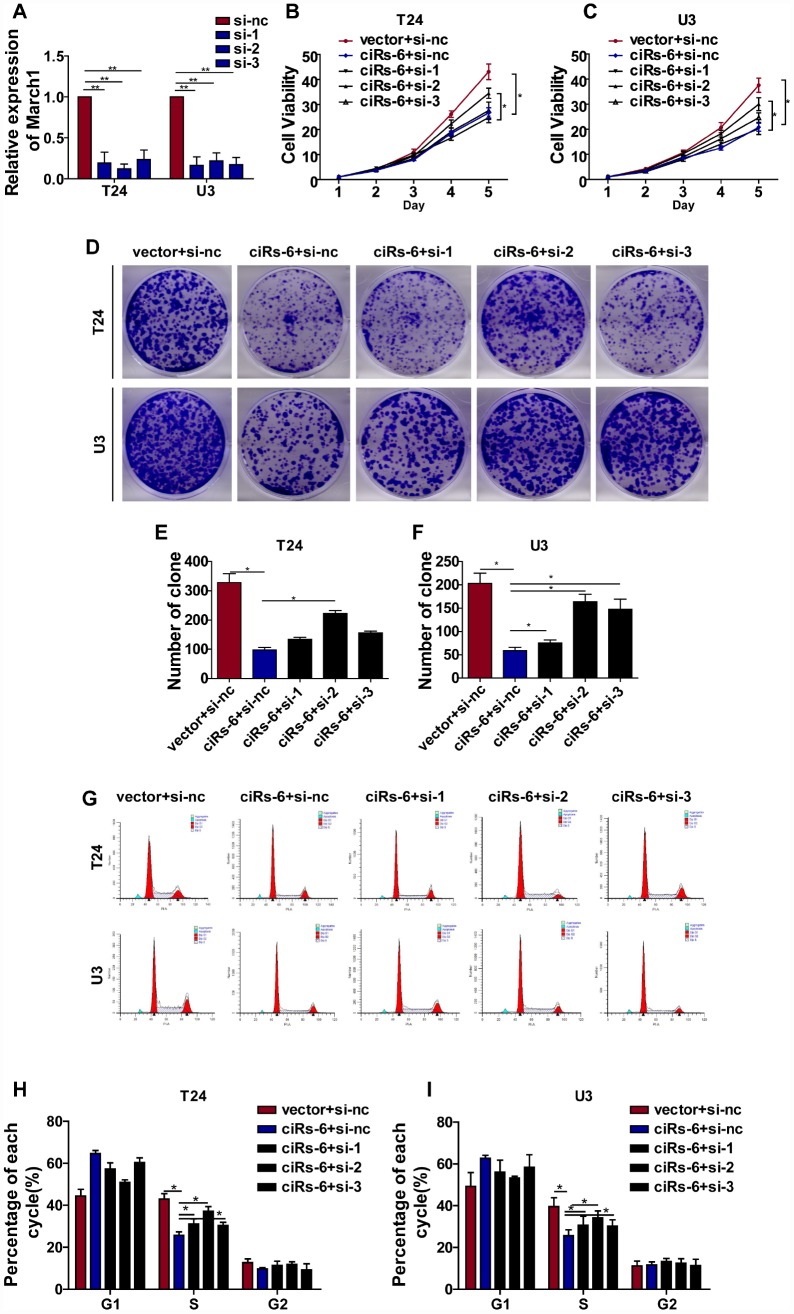
**ciRs-6 suppresses bladder cancer growth by elevating March1 levels**. Three siRNAs for March1 were designed and transfected into ciRs-6 overexpressed bladder cancer cells. (**A**) qPCR was used to detect the level of March1 in each group; (**B**, **C**) CCK8 assays were performed to evaluate cell viability; (**D**–**F**) clone formation assays were used to evaluate clone forming ability; (**G**–**I**) cells in S phase were assessed by cell cycle analysis in each group. The results are displayed as the mean±SEM, *p<0.05, **p<0.01.

Because ciRs-6 sponges miR-653, we used Targetscan, miRDB and RNAhybrid to predict the downstream targets of miR-653. Seventeen targets were identified by all 3 websites (Figure 5A). Then, we performed qPCR analysis to detect the mRNA levels of these 17 targets when ciRs-6 was overexpressed. March1 was found to be elevated by 40-fold in T24 cells and 31-fold in U3 cells when ciRs-6 was overexpressed (Figure 5B). This result suggested that March1 might be rescued by ciRs-6 from miR-653. Additionally, miR-653 was shown to reduce March1 levels at both the mRNA and protein levels (Figure 5C, 5G 5H); further, overexpression of miR-653 significantly reduced luciferase activity from the March1 reporter, which verified the interaction between March1 and miR-653 (Figure 5D, 5E). These results suggest that March1 is the direct downstream target of miR-653. Moreover, to verify the working axis of ciRs-6/miR-653/March1, when miR-653 was co-overexpressed with ciRs-6 in bladder cancer cells, the level of March1 was obviously decreased to a level that was similar to what was observed in wild-type cells (here, as shown in vector) (Figure 5F–5H). These results suggest that ciRs-6 sponging of miR-653 elevates March1 levels. To further verify that ciRs-6 suppressed bladder cancer growth by elevating March1 levels, we designed 3 siRNAs to silence March1 (Figure 6A). March1 expression was significantly silenced (Figure 6A), and there was little effect on ciRs-6 and miR-653 expression (Supplementary Figure 5A). Silencing March1 reversed the tumor suppressive role of ciRs-6 on bladder cancer cells, which resulted in improved cell viability (Figure 6B, 6C), higher clone formation ability (Figure 6D–6F) and a higher percentage of cells in S phase (Figure 6G–6I). In summary, these data indicate that the tumor suppressive role of ciRs-6 was driven by March1 elevation in bladder cancer.

### March1 suppresses bladder cancer growth in vitro and in vivo

To next determine the role of March1 in bladder cancer, stable overexpressed March1 bladder cancer cell lines were constructed, and the efficacy of overexpression was determined by qPCR (Supplementary Figure 4A). Overexpression of March1 significantly reduced bladder cancer cell viability (Supplementary Figure 4B, 4C), clone formation ability (Supplementary Figure 4D, 4E) and the percentage of cells in S phase (Supplementary Figure 4F–4I); moreover, an in vivo mouse model showed a positive tumor suppression effect when March1 was overexpressed (Supplementary Figure 4J–4L). Above all, the results suggest that March1 suppresses bladder cancer growth.

## DISCUSSION

As a result of deep sequencing and a novel bioinformatics approach, numerous circular RNAs have been identified. Unfortunately, few circular RNAs have been proved to play a role in driving cancer progression recently [3, 7, 11]. According to our previous RNA-seq analysis in 2 paired bladder cancer tissues, ciRs-6 was significantly downregulated in bladder cancer tissues compared to normal controls [10]. Clinical pathological analysis revealed that higher ciRs-6 levels negatively correlated with tumor grade, TNM stage and overall survival of bladder cancer patients. As demonstrated in multiple studies in either plasma or tissue, the varied expression of circular RNAs statistically correlated with different clinical features. Combined with the results of ciRs-6 shown here, the data suggest that ciRs-6 may serve as a potential biomarker to assist in cancer diagnosis and patient follow-up, as many people are reluctant to undergo invasive cystoscopy tests [12]. Hence, future studies should use larger sample cohorts and samples from different ethnic regions to further analyze the potential of ciRs-6 as a biomarker for bladder cancer.

Next, to detect the role of ciRs-6 in bladder cancer biology, in vivo and in vitro experiments were performed, and they revealed that ciRs-6 suppressed bladder cancer growth. As we mentioned before, cellular location indicates molecular function. Numerous studies suggest that cytoplasmic circular RNAs are inclined to posttranscriptionally regulate gene expression, particularly through the sponging of miRNAs [13]. Hence, we indicated that cytoplasmic localized ciRs-6 suppresses bladder cancer growth by sponging miR-653. Among multiple targets of miR-653, March1 expression was altered significantly after ciRs-6 was disrupted. Silencing March1 expression inversed the tumor suppressive role of ciRs-6 in bladder cancer. This suggests that March1 is the main downstream target of ciRs-6 in bladder cancer.

March1 is an E3 ubiquitin-ligating enzyme that has known functions in the immune system. As reported previously, March1 was highly expressed in APCs (antigen presenting cells) to prevent MHC class II recycling and promote lysosomal targeted degradation, thus blocking acute APC activation and reducing efficient antigen presentation to CD4 T cells [14, 15]. Although previous studies on March1 focused less on cancer biology, Ying Meng et al. suggested that March1 was overexpressed in ovarian cancer tissues compared with normal control tissues. Silencing March1 inhibits proliferation, migration and invasion of ovarian cancer cells by affecting the NF-κB and Wnt/β-catenin pathways [16]; Xie L et al. demonstrated that March1 promotes tumor progression of hepatocellular carcinoma via regulation of the PI3K-AKT pathway [17, 18]. This suggests that March1 plays a role in cancer biology and partially supports our findings regarding ciRs-6/miR-653-5p/March1 in bladder cancer. However, the underlying mechanisms in suppressing bladder cancer growth remain unclear.

Subsequently, to explore the potential mechanisms of March1 in bladder cancer, in vivo and in vitro experiments were performed. It was shown that, similar to ciRs-6, March1 mainly suppresses bladder cancer growth and has less of an effect on cancer migration and invasion. Considering that March1 is an E3 ubiquitin-ligating enzyme, it is important that future work determine whether March1 exerts its role of ubiquitination in suppressing bladder cancer.

## CONCLUSIONS

Overall, our study is the first to investigate the role of ciRs-6 in bladder cancer, not only as a potential diagnostic biomarker but also as a suppressor of bladder cancer growth. Additionally, this is the first research to explore the underlying mechanisms of March1 in bladder cancer biology. We hope these findings provide a better understanding of cancer biology, and we hope that a larger follow-up study will offer deeper understanding of the potential role of ciRs-6 as a diagnostic and prognostic biomarker for bladder cancer.

## MATERIALS AND METHODS

### Human tissue management

45 paired bladder cancer tissues and adjacent normal tissues and 58 bladder cancer tissues were obtained from patients diagnosed with bladder cancer at Sun Yat-Sen Memorial Hospital from 2014 Jun 1^st^ to 2019 Mar 1^st^. Only 43 patients were followed finally. The use of those tissues was approved by the ethics committee of Sun Yat-Sen University. All patients signed consent for the use of their tissues experimentally. All tissue samples were collected in liquid nitrogen and stably stored at -80°C until RNA extraction. Pathological and histological diagnoses of these samples were confirmed by 3 pathologists. All patients were followed up from the first diagnosis until death.

### Cell culture

Human bladder cancer cell lines T24 (RRID: CVCL_0554) and UM-UC-3 (U3, RRID: CVCL_1783) were purchased from ATCC. T24 cells were cultured in 1640 medium, and U3 cells were cultured in DMEM medium (Gibco, USA) with 10% FBS (Gibco, USA) and 100 U/ml penicillin and streptomycin (Gibco, USA) under a humidified atmosphere of 37°C and 5% CO2 (Thermo, Germany). All cell lines were validated by iGene Technology (Guangzhou, China) with STR assessments in the last 6 months.

### RNA preparation and quantitative real-time PCR

Total RNA was extracted by using a rapid RNA extraction kit (ES Science, China), and RNA was reverse-transcribed using a PrimeScript^TM^ RT Master Mix Kit (TakaRa, Japan). Quantitative real-time PCR was performed with a TB Green Premix Ex Taq II Kit (TakaRa, Japan) and analyzed by a Light Cycler 480 (Roche, German). Primers for qPCR are listed in Supplementary Table 1.

### Agarose gel electrophoresis and RNase R treatment

Detailed procedures of agarose gel electrophoresis and RNase R treatment are shown in our previously published paper [10].

### Florescent in situ hybridization (FISH)

FISH probes for ciRs-6 and miR-653 were synthesized by GenePharm (Suzhou, China). FISH was performed according to procedures provided by RiboBio (Guangzhou, China). 18S RNA was used as a positive control. The sequences for the FISH probes used in this study are listed in Supplementary Table 2.

### Nuclear and cytoplasmic extraction assay

Nuclear and cytoplasmic RNAs were extracted according to procedures provided by ThermoFisher (78833, German).

### Cell transfection

siRNAs for ciRs-6 and March1 were purchased from Genepharm (Suzhou, China). The exact procedures have been described previously. The sequences for the siRNAs used in this study are listed in Supplementary Table 3.

Lentivirus-containing plasmids for overexpression were constructed by iGene (Guangzhou, China). ciRs-6 was cloned into a plenty-ciR-GFP-T2A-puro vector, and miR-653 was cloned into a PCDH-CMV-MCS-EF1a-GFP-puro vector.

### CCK8 viability assay

Cells were seeded at a density of 1000 cells/well in a 96-well plate 24 h before the experiment. Then, 100 μl of medium containing 10% CCK8 (Beyotime, Suzhou, China) was added to each well, and OD values were measured at 452 nm after 2 h of incubation using a microplate reader.

### Clone formation assay

Cells were seeded at a density 1000 cells/well in 6-well plates in medium with 10% FBS. The medium was changed every 3 days. Clones were stained with 1% crystal violet when >50 cells were observed from one clone.

### Cell cycle stain and analysis

Cells were harvested and fixed with 70% ethanol for more than 12 h. The cells were stained and analyzed by flow cytometry according to the manufacturer’s instructions (F10797, Invitrogen, USA).

### Tumor subcutaneous mouse model

All animal studies were approved by The Animal Management Committee of Sun Yat-Sen University. A total of 10^7^ cells were injected subcutaneously into nude mice (3-4 weeks old, female). The weight (W) and length (L) of tumors were measured every week with calipers. Mice were sacrificed when the tumor volume reached over 200 mm^3^, and the volume was determined with the following equation: V=(L×W×W)/2. Tumor weight was measured when the mice were sacrificed. Harvested tumors were fixed in 4% polyformalin and embedded in paraffin for immunohistochemistry.

### Western blot analysis

Protein samples were extracted with RIPA buffer (Beyotime, Suzhou, China) containing 1% proteinase inhibitor (Roche, Germany). The concentrations of the protein samples were evaluated using a BCA assay kit (ThermoFisher, Germany). Detailed procedures were described previously. The antibody for March1 that was used in this study was purchased from HuosierM (China) and diluted at a concentration of 1:1000.

### Immunohistochemistry

Samples were collected from mouse subcutaneous tumor samples. The antibody for March1 used in this study was purchased from HuosierM (China) and was diluted at a concentration of 1:200. Each tissue sample slide was assessed by 3 pathologists, and the degree of positivity was determined by the percentage of positive cells.

### Dual luciferase reporter assay

Predicted binding sites for miR-653 in ciRs-6, and predicted binding sites for miR-653 in the 3′UTR of targets of miR-653 were cloned into a dual luciferase reporter vector, psi-check2, by Synbio Technologies (Suzhou, China). The constructed vectors were then transfected with miR-653 using Lipofectamine RNAiMAX reagent (Invitrogen, USA). After 48 h of transfection, cells were harvested, and luciferase activity was measured according to the manufacturer’s protocol (E292, Promega, USA).

### RNA pulldown

For pulling down miRNAs with a circular RNA, a specific biotin-labeled probe for ciRs-6 was designed and synthesized by Genepharm (Suzhou, China). The ciRs-6 probe and the oligo probe were incubated with 25 μl of streptomycin-magnetic beads for 2 h and were incubated with shaking at room temperature. Then, 10^7^ cells were harvested and lysed in RIPA buffer. Cell lysates were incubated with probe-coated beads for more than 12 h, and the RNA complex that was bound to the magnetic beads was washed and separated by PBS and a magnetic separator. RNA in the isolated complex was then extracted, and qPCR was used for miRNA detection.

For circular RNAs pulled down by miRNA, biotin-labeled miR-653 mimics were designed and synthesized by Genepharm (Suzhou, China). Cells were transfected with miR-653 mimics for more than 48 h, and 10^7^ cells were harvested and lysed in RIPA buffer. Cell lysates were incubated with streptomycin-magnetic beads for 12 h, and RNA complexes that bound to the magnetic beads were washed and separated by PBS and a magnetic separator. RNA in the isolated RNA complex was extracted, and qPCR was used for circular RNA detection. The probe sequences used in this study are listed in Supplementary Table 4.

### Statistical analysis

Statistical analysis was carried out using GraphPad 5.0. Statistical significance between 2 independent groups was determined using t-tests. One-way ANOVA was used to evaluate significance among multiple groups. Kaplan-Meier curves and log-rank tests were used for survival analysis. P<0.05 was considered statistically significant.

### Ethics approval

The use of human tissues was approved by the ethics committee of Sun Yat-Sen University. All animal studies were approved by The Animal Management Committee of Sun Yat-Sent University. All patients signed the consent for using their tissues experimentally.

## Supplementary Material

Supplementary Figures

Supplementary Tables
