# Correction for: ciRs-6 upregulates March1 to suppress bladder cancer growth by sponging miR-653

**DOI:** 10.18632/aging.102885

**Published:** 2020-03-02

**Authors:** Yinjie Su, Weilian Feng, Guanglei Zhong, Yiyao Ya, Zehu Du, Juanyi Shi, Luping Chen, Wen Dong, Tianxin Lin

**Affiliations:** 1 The Department of Urology, Sun Yat-Sen Memorial Hospital, Sun Yat-Sen University, Guangzhou, China; 2 The Department of Endocrinology, Sun Yat-Sen Memorial Hospital, Sun Yat-Sen University, Guangzhou, China; 3 The Department of Gynecological oncology, Sun Yat-Sen Memorial Hospital, Sun Yat-Sen University, Guangzhou, China; 4 The Department of Urology, Guangzhou First People's Hospital, School of Medicine, South China University of Technology, Guangzhou, China; 5 The Department of Thyroid Surgery, Sun Yat-Sen Memorial Hospital, Sun Yat-Sen University, Guangzhou, China; 6 The Department of Pediatric Surgery, Sun Yat-Sen Memorial Hospital, Sun Yat-Sen University, Guangzhou, China

**Keywords:** correction

**This article has been corrected:** The authors requested the replacement of Figure 4E and Table 1. Figure 4E (ciRs-6+miR-nc) was quite similar to Supplementary Figure 1D (si-3 in T24 cells) and was slightly updated. Changes of Table 1 are done to correct the statistical mistakes.

These corrections do not change the content of the publication and do not affect the conclusion of this research. The authors apologize for the unintentional mistakes.

The corrected Figure and Table are provided below.

**Figure 4:**
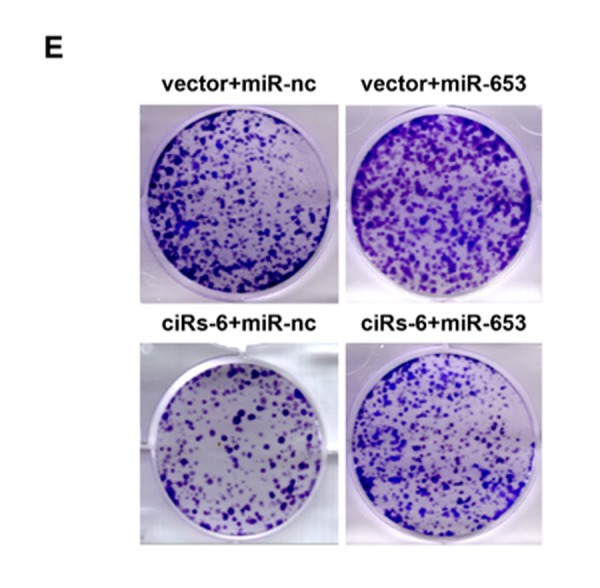
**miR-653 rescues the tumor suppressive effect of ciRs-6 in bladder cancer.** (**E**) Clone formation assays were used to evaluate clone forming ability.

**Table 1:** Relationship between ciRs-6 level and clinical characteristics in bladder cancer.

**Total**		**Patients**	**Expression of ciRs-6**		
			**High**	**Low**	**p**
**Age(mean)**		50	47.931	52.069	0.395
**Gender**					
	Male	45	22	23	0.753
	Female	13	7	6	
**Tumor stage**					
	Tis/Ta/T1	31	22	9	<0.001
	T2	17	7	10	
	T3/T4	10	0	10	
**Grade**					
	High	20	5	15	0.006
	Low	38	24	14	
**Number of tumors**					
	Solitary	41	23	18	0.149
	Multiple	17	6	11	
**Lymph node metastasis**					
	Negative	23	13	10	0.421
	Positive	35	16	19	
**Follow-up (month, mean)**		33.23	33.00	33.45	0.047

Original article: Aging. 2019; 11:11202–11223.  https://doi.org/10.18632/aging.102525

