# Supplementary material for: ciRs-6 upregulates March1 to suppress bladder cancer growth by sponging miR-653
Source: Aging (Albany NY). 2019 Dec 10;11(23):11202–23. doi: 10.18632/aging.102525 (PMC6932879; doi:10.18632/aging.102525)
Supplement: Supplementary Figures [file aging-11-102525-s002..pdf]

SUPPLEMENTARY FIGURES

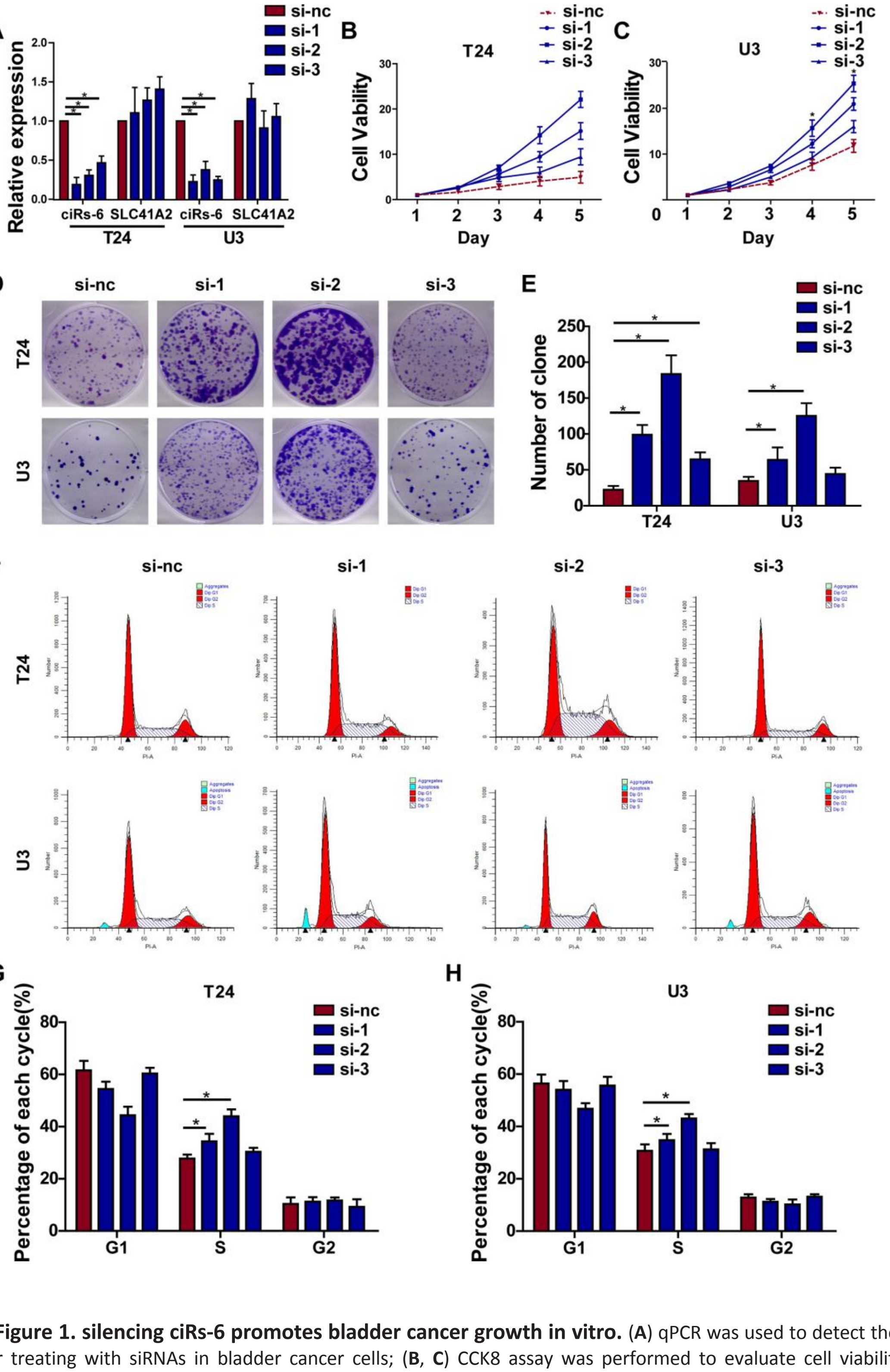

Supplementary Figure 1. silencing ciRs-6 promotes bladder cancer growth in vitro. (**A**) qPCR was used to detect the level of ciRs-6 and SLC41A2 after treating with siRNAs in bladder cancer cells; (**B**, **C**) CCK8 assay was performed to evaluate cell viability; (**D**, **E**) clone formation assay was used to evaluate clone forming ability; (**F**, **G**) S phage of cells were determined by cell cycle analysis. Results are displayed as mean±SEM, *p<0.05, **p<0.01.

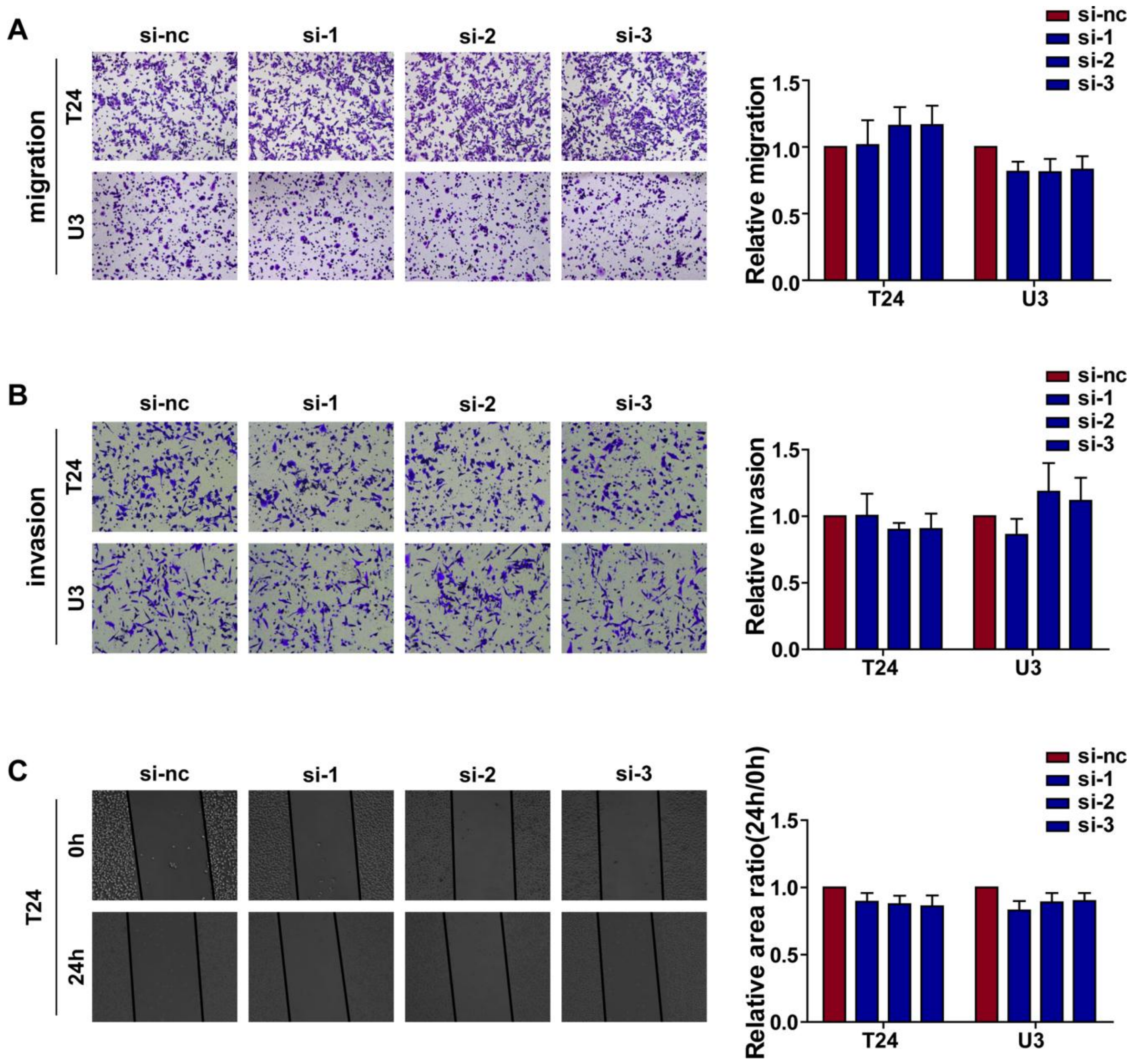

Supplementary Figure 2. Silencing ciRs-6 leads less effect on bladder cancer cell metastasis in vitro. Invasion and migration of bladder cancer cells was determined in vitro by (**A**, **B**) Trans-well assay; and (**C**) wound healing assay. Pictures were photographed by 200×under light microscope. Results are displayed as mean±SEM, *p<0.05, **p<0.01.

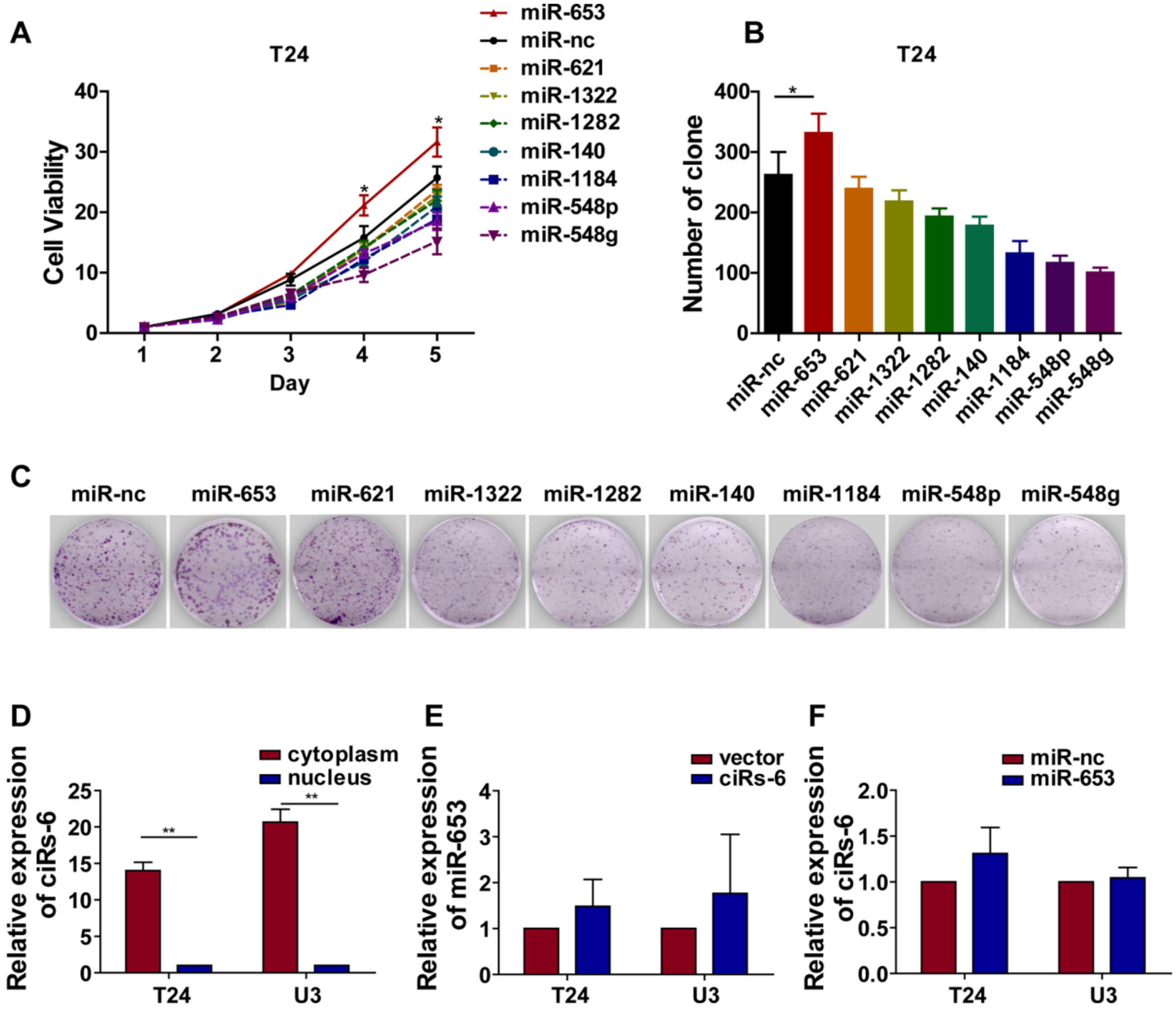

Supplementary Figure 3. Effect of 8 miRNAs on cell growth in vitro. (**A**) CCK8 assay used to detect the cell viability among each miRNA treating group; (**B**, **C**) showed different clone forming ability after miRNAs over-expression; (**D**) location of cirRs-6 in cells was determined by its different level in cytoplasm and nucleus; (**E**) expression of miR-653 was detected after ciRs-6 over-expression; (**F**) expression of ciRs-6 was detected after miR-653 over-expression. Results are displayed as mean±SEM, *p<0.05, **p<0.01.

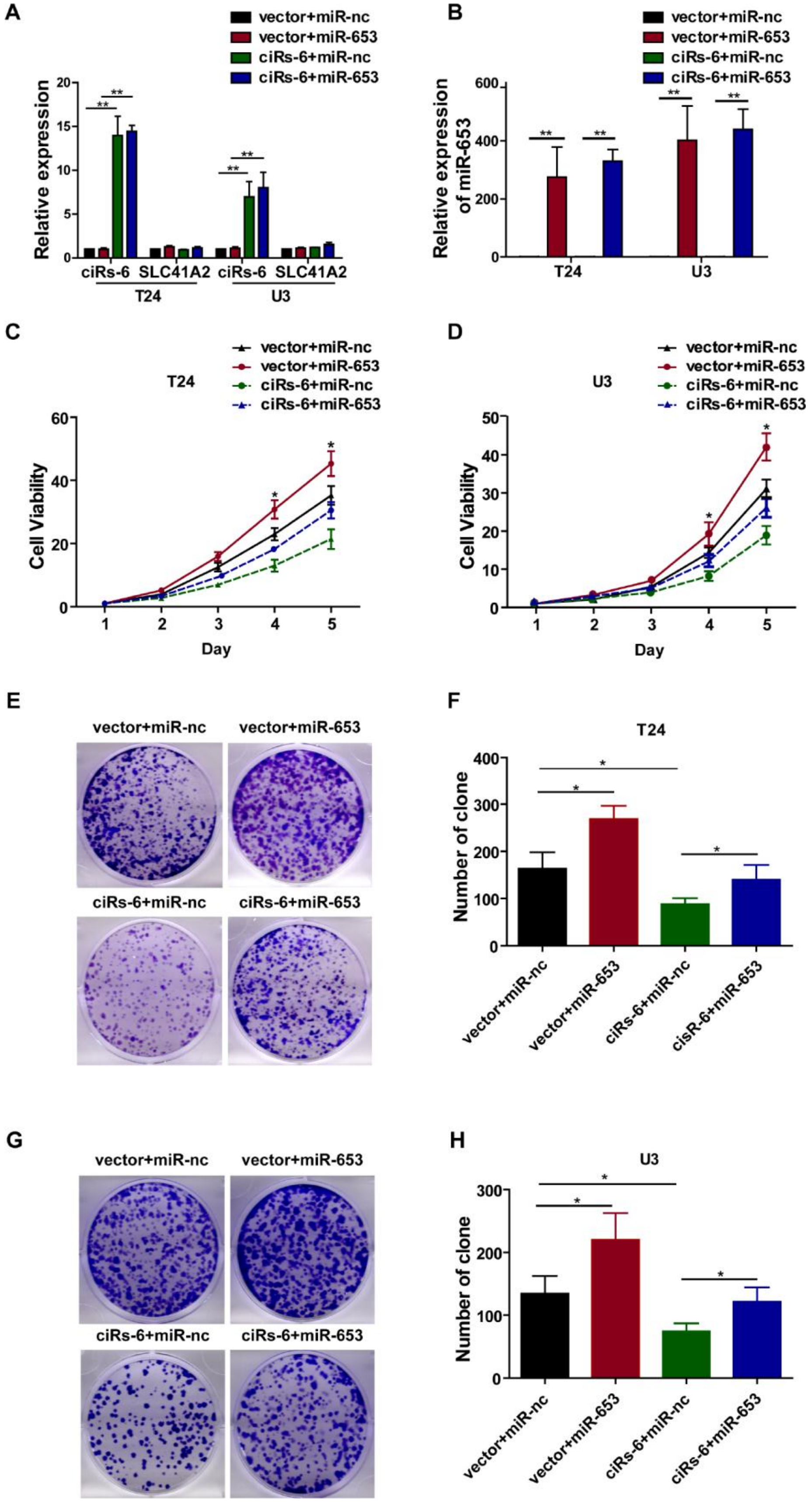
A
vector+miR-nc
vector+miR-653
ciRs-6+miR-nc
ciRs-6+miR-653
Relative expression
ciRs-6 SLC41A2 ciRs-6 SLC41A2
T24
U3
B
Relative expression of miR-653
T24
U3
C
T24
Cell Viability
Day
D
U3
E
F
Number of clone
vector+miR-nc
vector+miR-653
ciRs-6+miR-nc
cisR-6+miR-653
G
ciRs-6+miR-653
H
U3

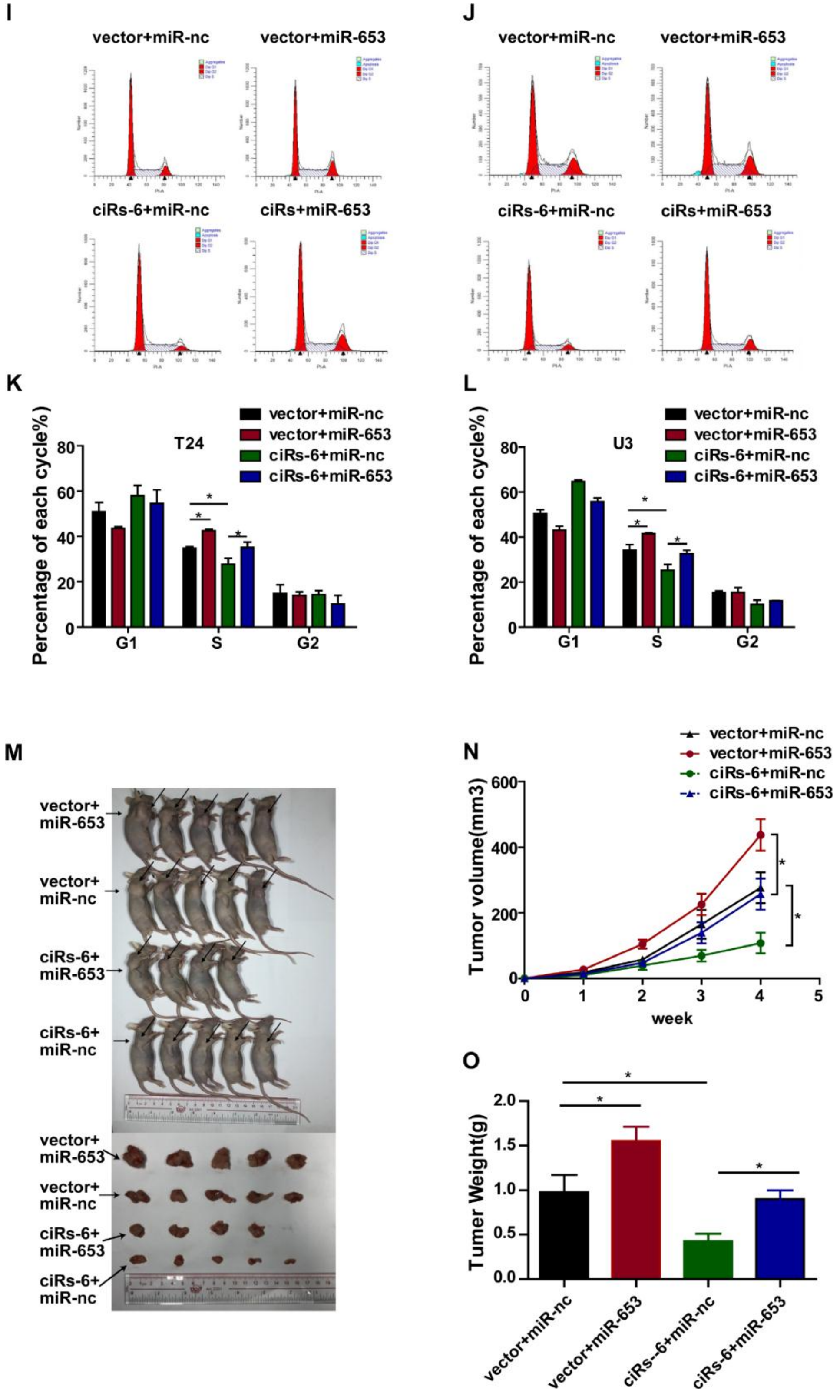

Supplementary Figure 4. overexpressing March1 suppresses bladder cancer growth in vitro and in vivo. (**A**) level of March1 was determined by qPCR; (**B**, **C**) CCK8 assay was performed to evaluate cell viability; (**D**, **E**) clone formation assay was used to evaluate the ability to form clones; (**F**–**I**) S phage of cells were determined by cell cycle analysis; (**J**–**L**) mice subcutaneous tumor model was involved to detect the suppressive role of March1 in tumor growth in vivo. Each group contains 5 mice. Results are displayed as mean±SEM, *p<0.05, **p<0.01.

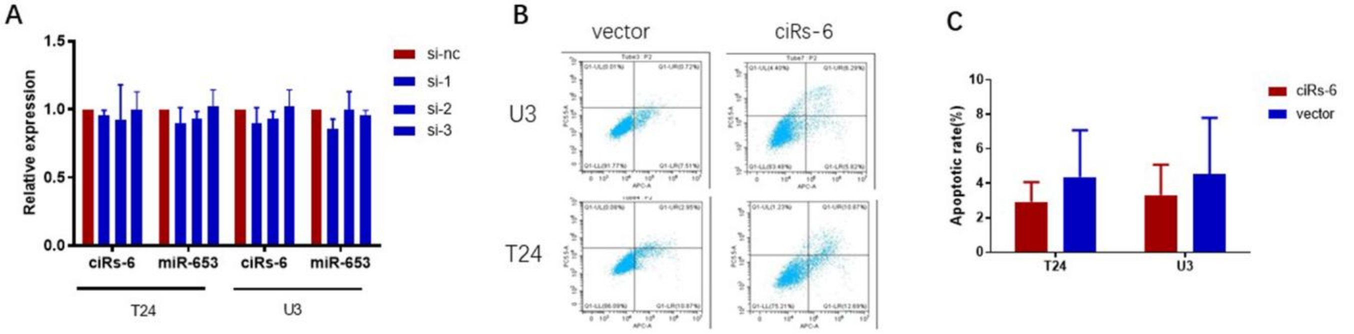

Supplementary Figure 5. overexpressing March1 suppresses bladder cancer growth in vitro and in vivo. (**A**) Expression of ciRs-6 and miR-653 were determined after March1 silence; (**B**, **C**) Annexin V/PI assay shown that over-expressing ciRs-6 in bladder cancer cells leads less effect on bladder cancer cell apoptosis. Results are displayed as mean±SEM, *p<0.05, **p<0.01.
