# Supplementary material for: ciRs-6 upregulates March1 to suppress bladder cancer growth by sponging miR-653
Source: Aging (Albany NY). 2019 Dec 10;11(23):11202–23. doi: 10.18632/aging.102525 (PMC6932879; doi:10.18632/aging.102525)
Supplement: Supplementary Tables [file aging-11-102525-s001..pdf]

SUPPLEMENTARY TABLES

Supplementary Table 1. List of primers for qPCR.

Gene	Sequence (5′-3′)
ciRs-6	F: CAGTTTCAGCTGGCATGGTA<br>R: TGAATCAGAACCGCACAAAC
SLC41A2	F: AATGGTTGGGGTTATCGTTG<br>R: TTCTTGTGGCTGGATGTTTG
GAPDH	F: TACTAGCGGTTTTACGGGCG<br>R: TCGAACAGGAGGAGCAGAGAGCGA
miR-653-5p	F: CTCAACTGGTGTCGTGGAGTCGGCAATTCAGTTGAGCAGTAGAG<br>R: ACACTCCAGCTGGGGTGTTGAAACAATCT
March1	F: GTCTGTCCATCCACTCAGGAC<br>R: GGTTTGAGCTTGGTCTCCATT
U6	F: CGCTTCGGCAGCACATATAC<br>R: TTCAGAATTTGCGTGTCAT

Supplementary Table 2. List of sequences for FISH probe.

Gene	Sequence
ciRs-6	Cy3-GTTCCACAGAAGGACTGGATCTGTACTATATCCAGTACCA
miR-653-5p	Cy5-CAGATGAGATTGTTTCAACAC
18S RNA	Cy5-CTTCCTTGGATGTGGTAGCCGTTTC
U6	Cy5-TTTGCGTGTCATCCTTGCG

Supplementary Table 3. List of siRNAs.

siRNA	Sequence (5′ -3′)
ciRs-6 si-1	sense: UGGAUAUAGUACAGAUCCATT<br>antisense: UGGAUCUGUACUAUAUCCATT
ciRs-6 si-2	sense: AUAGUACAGAUCCAGUCCUTT<br>antisense: AGGACUGGAUCUGUACUAUTT
ciRs-6 si-3	sense: GUACAGAUCCAGUCCUUCUTT<br>antisense: AGAAGGACTGGATCTGTACTT
March1 si-1	sense: CAGCCACGUUUGUUGUAAUTT<br>antisense: AUUACAACAAACGUGGCUGTT
March1 si-2	sense: GAUGCCAAAUUAUCUAACUTT<br>antisense: AGUUAGAUAAUUUGGCAUCTT
March1 si-3	sense: CGUACAGUGUAAAGUCUAUTT<br>antisense: AUAGACUUUACACUGUACGTT

Supplementary Table 4. List of probe sequences for RNA pulldown.

Gene	Sequence (5′-3′)
ciRs-6 (5’biotin)	UCCACAGAAGGACUGGAUCUGUACUAUAUCCAGUACC
oligo	UUGUACUACACAAAAGUACUG
miR-653-5p (5’biotin)	GUGUUGAAACAAUCUCUACUG
	GUAGAGAUUGUUUCAACACUU
miR-nc (5’biotin)	UUCUCCGAACGUGUCACGUTT
	ACGUGACACGUUCGGAGAATT
